# A Mexican Spanish Adaptation of the Dutch Eating Behavior Questionnaire: Psychometric Properties and Influence of Sociodemographic Variables in Pregnant Women

**DOI:** 10.3390/nu15143243

**Published:** 2023-07-21

**Authors:** María Eugenia Flores-Quijano, Cecilia Mota-González, Guadalupe Rozada, Jacqueline Citlalli León-Rico, María Eugenia Gómez-López, Rodrigo Vega-Sánchez

**Affiliations:** 1 Department of Nutrition and Bioprogramming, Instituto Nacional de Perinatología, Ciudad de México 11000, Mexico; maru_fq@yahoo.com (M.E.F.-Q.); jaki.leon94@gmail.com (J.C.L.-R.); 2 Department of Psychology, Instituto Nacional de Perinatología, Ciudad de México 11000, Mexico; ceciliainper@gmail.com (C.M.-G.); eugeniagomez2712@yahoo.com.mx (M.E.G.-L.); 3 Private Consultant, Alimentación Plena, Ciudad de México 06760, Mexico; alimentacion.plena@gmail.com

**Keywords:** eating behaviors, DEBQ, restrained eating, emotional eating, external eating, pregnancy, psychometric properties, psychosocial factors

## Abstract

Eating behaviors are complex phenomena, entangling physiological signals of hunger and satiety, food choices, emotional states, and social factors and expectations, as well as food availability and sensory appearance. Evaluating eating behaviors is challenging and must cover different motives. One instrument for such evaluation is the Dutch Eating Behavior Questionnaire (DEBQ), composed of three subscales for exploring emotional eating, external eating, and restrained eating. In this article, we aimed to (1) evaluate the psychometric properties of a Mexican Spanish adaptation of the DEBQ; and (2) explore the associations between the three adapted DEBQ scales and the influence of sociodemographic factors on each of the three eating behaviors in Mexican pregnant women. A sample of 514 pregnant women responded to our adapted version of the DEBQ and a questionnaire about sociodemographic information. We performed an exploratory factor analysis using a principal component analysis with varimax rotation; based on this analysis, we removed items that loaded on two factors and then performed a confirmatory factor analysis. The final version of the adapted DEBQ has 26 items, clearly divided into a three-factor structure and satisfactorily reliable (Cronbach’s ⍺ = 0.903). We then performed Spearman bivariate correlations and multivariate linear regression with backward variable selection to test the associations and influence of sociodemographic factors on each of the three eating behaviors evaluated with the DEBQ. In pregnant women, emotional eating (EmoE) had a medium-high correlation with external eating (ExtE) and a low correlation with restrained eating (RestE), while ExtE and RestE showed no association. The three eating behaviors are associated with maternal sociodemographic and reproductive variables, which partly explain their variation, most notably maternal schooling. Our adapted version of the DEBQ is suitable for use with Mexican Spanish–speaking pregnant women. Maternal sociodemographic and reproductive factors have an influence on the variance of eating behaviors during pregnancy.

## 1. Introduction

An individual’s eating behavior is a very complex phenomenon. In addition to physiological signals of hunger and satiety, the choice and consumption of specific foods and their quantity are influenced by many other compelling reasons. These may include personal preferences, positive or negative emotional states, social motives including gatherings, norms, and expectations, as well as external cues that have to do with food availability, such as its taste, smell, and appearance [1,2].

Since the evaluation of eating behaviors is complex and challenging, the instruments used for their evaluation must cover different eating motives. One such instrument is the Dutch Eating Behavior Questionnaire (DEBQ) developed during the 1980s and composed of three scales sustained by three theories about eating [3]. The emotional eating (EmoE) scale is based on the psychosomatic theory, which proposes that emotional arousal states such as anger, fear, and anxiety elicit eating as a learned mechanism to cope with or reduce the negative state. The external eating (ExtE) scale, based on the externality theory, measures the tendency to eat in response to external food-related cues such as the sight, taste, and smell of attractive food, a “heightened sensitivity to food cues” regardless of the internal signals of hunger and satiety. The restrained eating (ResE) scale is based on the homonym theory and measures how much individuals consciously restrict their food intake (i.e., dieting). Intense restriction, a response to social pressure, may result in persistent hunger, overeating, anxiety, or depression, thus triggering emotional and external eating behaviors [3,4].

Since its development, the DEBQ has been translated into several languages, Spanish among them [5]. The latter adaptation, however, only included women from Spain and, while the authors “took great care to use a standard version of Spanish language so it could be also well understandable for Latin-American people” [5], its use may still not be suitable for use with Latin American populations due to linguistic nuances and, particularly, cultural differences. Moreover, even if the Spanish version of the DEBQ were completely applicable in Latin America, still, a particular and prevalent population was not included in its development: pregnant women.

The complete DEBQ or one or two of its scales have been used in several studies with pregnant women. Studies conducted in developed countries such as the UK [6], the USA [7,8], and Israel [7] used the English version of the questionnaire. Other studies have been done in Iran [9], where women self-responded to a Persian-translated questionnaire, and in China [10], where a Chinese version of the instrument had been validated in adolescents [11].

These studies have described different ways in which pregnancy modifies DEBQ-measured eating behaviors. For example, some women experience lower levels of restrained eating compared to their non-pregnant state [6], while cravings may mediate the association between EmoE and excessive gestational weight [8]. Moreover, changes in eating behaviors vary with respect to cultural background, as shown by a comparison between Israeli and British women [7]. Despite these findings, the psychometric properties of the DEBQ have not been formally validated in pregnant women.

Therefore, in this study, we aimed to (1) evaluate the psychometric properties of a Mexican Spanish translation and adaptation of the original DEBQ, and (2) examine correlations between the three eating behavior scales and between each scale and sociodemographic characteristics.

## 2. Materials and Methods

### 2.1. Translation and Adaptation

The English version of the DEBQ, as it appears in the original publication [3], was translated into Spanish and adapted to Mexican cultural terminology by two nutrition specialists (GR and RVS). An external bilingual nutrition specialist performed a back-translation, ensuring that the original meaning of each item was not modified during translation/adaptation. Our translated and adapted version could not be included in this paper due to copyright restrictions of the original scale.

We applied our adapted translation of the DEBQ to a group of pregnant women who received their prenatal care at the National Institute of Perinatology (INPer) in Mexico City and gave their informed consent to participate. The study was approved by INPer’s Research and Ethics Committees (Reg. No. 2018-1-169).

### 2.2. Evaluation of Psychometric Properties

#### 2.2.1. Subjects and Procedures

Our study included a convenience sample of 514 pregnant women. The rationale for this sample size was that a confirmatory factor analysis requires a minimum of 10 cases (responses) per each item in the questionnaire. Since the original DEBQ consists of 33 items, a minimum of 330 participants were needed. We decided to go well beyond this requirement and included 514 participants, thus assuring the statistical reliability of our analyses.

In order to apply our adapted version of the DEBQ to participating women, we included the adapted questionnaire’s items in an online form using Google Forms to ensure that all items were responded to. The answers were securely cloud-stored in a spreadsheet associated with a dedicated account. We sent a link to the form to each participant, who answered the questionnaire online.

Along with the DEBQ, the online form also asked participants to provide their age, number of pregnancies (including the current one), number of miscarriages/stillbirths, current gestational week, type of pregnancy (single/multiple), current illness, highest school degree, occupation (stays at home/works outside the home), whether they lived with the baby’s father, their household welfare, height, and pregestational weight. From the last two data, we calculated each woman’s pregestational body mass index (pg-BMI).

Current illness refers to having any medical condition, whether acute (e.g., infections) or chronic (e.g., diabetes, autoimmune disease, thyroid conditions, preeclampsia). Household welfare was estimated using the AMAI rule 8 × 7, a tool developed by the AMAI (in Spanish, Mexican Association of Market Intelligence and Public Opinion Agencies). It categorizes households on seven socioeconomic levels (A/B, C+, C, C−, D+, D, and E) according to the head of the household’s ability to satisfy its members’ needs [12]. We grouped these seven categories into medium to high (C+, C, C−, A/B) and low (D+, D, E) household welfare.

From the online spreadsheet containing the participants’ answers, we constructed a database where participants were anonymized by assigning an alphanumeric ID to each. Only the PI (RVS) had access to the participant’s personal information, which was not shared with the rest of the team. We used this database to perform the analyses detailed next.

#### 2.2.2. Adaptation Quality, Reliability, and Validity Tests

To determine the quality and reliability of each of the translated items and eliminate those that had weak technical properties, for each item we obtained descriptive measures, frequency distributions, and Spearman correlations. We also performed Student *t*-tests for independent samples, between subjects who scored low on the total scale (below quartile 1) and those with the highest scores (above quartile 3), to discriminate between extreme groups. *p* values ≤ 0.05 were considered significantly different.

We then performed an exploratory factor analysis for the initial 33 translated items using a principal component analysis with varimax rotation. After removing items that loaded on two factors simultaneously (see Results section), we analyzed the overall instrument reliability using the Cronbach alpha test for the final 26 items.

#### 2.2.3. Confirmatory Factor Analysis

Finally, based on the results from the exploratory factor analysis and after making appropriate modifications, we performed a confirmatory factor analysis using AMOS version 23. As indicators of the model’s fit, we evaluated: (1) Chi-square value (CMIN), which should be between 1 and 3, and not significant, for the model to be considered satisfactory; (2) Goodness of Fit Index (GFI), values should be close to 1 to show a very good fit; (3) baseline Comparative Fit Index (CFI), values should also be close to 1; and (4) Root Mean Square Error of Approximation (RMSEA), where values ranging from 0.05 to 0.08 are considered acceptable, and values ≤ 0.05 are considered excellent [13].

### 2.3. Associations with Sociodemographic Variables

To analyze the association between the three DEBQ-measured eating behavior scores, with the sociodemographic and reproductive variables, we performed Spearman bivariate correlation tests. Subsequently, a multivariate linear regression using the backward variable selection procedure (F significance values: entry 0.05 and removal 0.10) was carried out using each eating behavior as the dependent variable in order to explore how much these variables explain the variance of the eating behaviors. The collinearities between those variables included in the final models were within accepted values: tolerance ≥ 0.926 and variance inflation factor ≤ 1.080.

All statistical analyses were carried out in SPPS version 26 considering *p*-values ≤ 0.05 as statistically significant.

## 3. Results

### 3.1. Evaluation of Psychometric Properties

#### 3.1.1. Participants’ Characteristics

Table 1 shows the sociodemographic and reproductive characteristics of our study sample. Participants’ median age was nearly 30 years, with a median pregestational BMI of 25.9 kg/m^2^). Most of them had singleton pregnancies (94.5%) and were multiparas in their second/third (42%) or on their fourth or more (19.3%) gestation. A high proportion of women (46.1%) reported having a disease that increased the risk for a complication of pregnancy, such as diabetes, thyroid illness, hypertension, or autoimmune disease, or already had a complication that increased the risk for negative results of her pregnancy, like preeclampsia, gestational diabetes or cervical incompetence, to name a few. Many participants had experienced at least one previous abortion or stillbirth (37.7%). The majority had at most achieved high-school education (70.7%), stayed at home (68.9%), lived with their baby’s father (64%), and lived in a low welfare household (74.3%).

#### 3.1.2. Adaptation Quality, Validity, and Reliability Tests

From participants’ answers, we first obtained descriptive measures for each item’s responses, to determine their quality and viability, and to eliminate those with weak technical properties. The 33 original items had normally distributed response frequencies, with similar means close to the theoretical mean, and relatively consistent and similar standard deviations (Table 2). The range of obtained responses covered the full range of possible answers on the Likert scale (1 to 5).

We then obtained bivariate correlations between all items. Most of them had correlation coefficients < 0.20, indicating that they were unrelated and, therefore, did not evaluate the same construct. Some items were correlated, but we decided not to eliminate any of them at this point (Table 3). Additionally, the comparative analysis to discriminate between extreme groups, i.e., lowest scores on the total scale (<Q1, n = 138, 26.8%) vs. highest scores (>Q3, n = 121, 23.5%), showed that all items were significantly different (*p* < 0.001), confirming that they adequately discriminated between response groups.

To obtain construct validity, we carried out an exploratory factor analysis (principal components with varimax rotation) for the 33 items of the instrument. This analysis showed a sample adequacy value of KMO = 0.914, and a statistically significant Bartlett sphericity value (X^2^_(325)_ = 8592.1, *p* < 0.001). Seven items (i4, i12, i23, i30, i31, i32, and i33) were eliminated because they loaded on two factors simultaneously, which resulted in an instrument comprised of 26 reagents. After this, the factor analysis converged in five iterations, yielding three factors with eigenvalues > 1 that explained 68.8% of the total variance, similar to the Italian [14] version of the DEBQ. These factors correspond to the three dimensions measured by the English version of the DEBQ scale: restrained eating (ResE), emotional eating (EmoE), and external eating (ExtE) (Table 4).

#### 3.1.3. Confirmatory Factor Analysis

**Figure 1:**
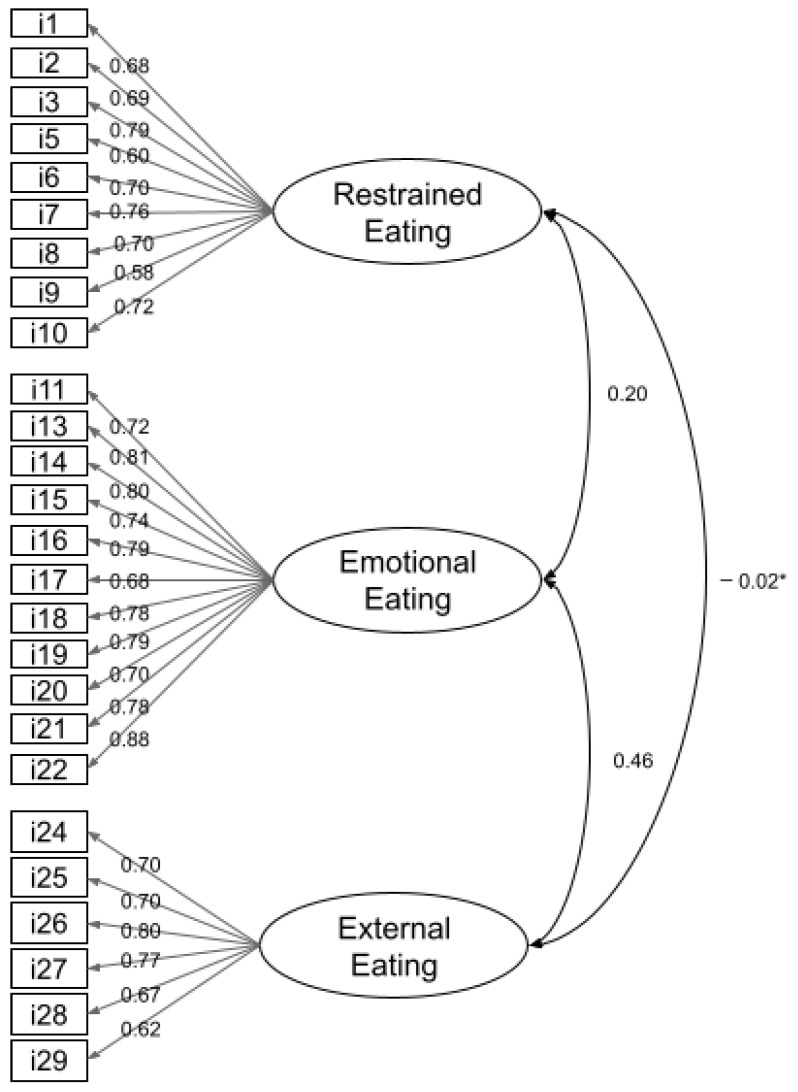
Factor loadings of the 26 items in the adapted DEBQ. * *p* not significant.

To analyze the questionnaire’s reliability, we then performed a reliability analysis for the remaining 26 items, which showed a Cronbach value of α = 0.903 for the entire questionnaire; 0.894 for the ResE subscale, 0.943 for the EmoE subscale, and 0.873 for the ExtE subscale. These reliability values are very similar to the original instrument [3,5]. Finally, a confirmatory factor analysis corroborated the same 26-item structure of the exploratory analysis, showing a good model fit: CMIN = 2.235; GFI = 0.917; CFI = 0.959; RMSEA = 0.049, *p* = 0.610. The 26 items had significant loads on their respective factors with standardized parameters ranging from 0.58 to 0.88 (Figure 1).

Factors for restrained and external eating showed little error covariance, while the emotional eating factor showed greater error covariance. However, since such errors were not significant, we did not eliminate any other item. The final DEBQ adapted to Mexican Spanish is composed of 26 items.

### 3.2. Associations and Regression Analyses between Eating Behaviors and Sociodemographic and Reproductive Variables

The correlations among the three eating behaviors are shown in Table 5. EmoE and ExtE scores were significantly associated (r = 0.626, *p* < 0.001). ResE was also associated with EmoE (r = 0.326, *p* = 006) but not with ExtE (r = 0.194, *p* = 0.110).

Also in Table 5, we show the bivariate correlations between each eating behavior and the sociodemographic and reproductive variables. Table 6 includes the results of the multivariate models.

ResE was significantly correlated with maternal age, pregestational BMI, number of pregnancies, occupation, and socioeconomic status. However, when all variables were entered into a multivariate regression model (Table 6), only maternal age (β = 0.030, *p* < 0.001), pregestational BMI (β = 0.045, *p* < 0.001), and schooling (β = 0.134, *p* < 0.001) explained a small proportion (r^2^ = 0.205, F(70, 272) = 43.747, *p* < 0.001) of the variance of the ResE behavior.

EmoE correlated with maternal age and schooling. However, in the multivariate analysis, age was found to be a confounding variable, since its association with EmoE was neutralized when variables like current illness (β = 0.173, *p* = 0.018) and the number of pregnancies (β = 0.085, *p* = 0.090) were included in the model. These two variables, together with schooling (β = 0.220, *p* < 0.001) explained a very small proportion (r^2^ = 0.065, F(3, 509) = 11.735, *p* < 0.001) of EmoE behavior.

As expected, having a previous miscarriage or abortion was highly associated with the number of pregnancies (r = 0.687, *p* < 0.001). We may propose that having a previous pregnancy loss may explain why the number of pregnancies has an effect on EmoE.

In both bivariate and multivariate analyses, ExtE was significantly correlated and explained by pregestational BMI (β = −0.013, *p* = 0.027), gestational weeks (β = 0.100, *p* = 0.060), and schooling (β = 0.104, *p* = 0.007). The r^2^ was extremely small (r^2^ = 0.030, F(3, 509) = 5.178, *p* = 0.002).

## 4. Discussion

In this study we describe the psychometric properties of an adaptation of the DEBQ into Mexican Spanish and the influence of sociodemographic and reproductive variables on eating behaviors in pregnant women.

Our Mexican Spanish adaptation of the DEBQ has important advantages over other existing adaptations. First, it includes cultural adaptations and linguistic nuances of Latin American Spanish, which makes it more appropriate for such populations, particularly Mexican individuals. Second, its psychometric structure and reliability were tested in pregnant women, a specific population that was neither included in the development of the original instrument nor in its first Spanish adaptation [5].

Regarding the instrument’s structure, unlike the original scale’s 33 items [3], our Mexican Spanish adaptation is composed of 26 items, grouped into the three original factors of the scale: ResE, EmoE, and ExtE. Seven items were eliminated from the original scale after the exploratory factor analysis since they showed factorial loads distributed into two factors. These were items 4, 12, 23, 30, 31, 32, and 33.

The first of these (item #4) is related to ResE, while items #12 and #23 are related to EmoE, and items #30 to #33 to ExtE. While these items measure some aspects of their respective eating behavior, our psychometric analyses showed that their elimination did not affect the instrument’s internal structure and capacity to discriminatively measure each behavior.

The instrument’s structure was corroborated with confirmatory factor analysis, obtaining a satisfactory criterion validity index, which indicates that the Mexican Spanish adaptation of the DEBQ for pregnant women measures the same constructs as the original version. Furthermore, this structure has been consistent in other studies where this instrument was used [5,15,16].

Regarding the instrument’s reliability, internal consistency values are adequate according to the indices proposed by DeVellis and Thorpe and Kaplan et al. [17,18]. In addition, the Cronbach’s alpha obtained for the total scale (0.903) is similar to that obtained in the original instrument [3], indicating that our adaptation’s results were highly satisfactory.

With respect to correlations among the three eating scales, our results replicate previous observations. The medium-high association between EmoE and ExtE scales has been revealed in several studies with diverse populations, such as female students without and with overeating problems [4,5], older women attending a health clinic [19], or candidates for bariatric surgery [20]. According to the theories underlying the scales, such a relationship is to be expected, since the three eating behaviors are intertwined regarding some assumptions: the externality theory considers emotionality as a manifestation of the general externality trait; in turn, in order for food consumption to occur in response to an emotional state, the presence and accessibility of foods that evoke the external stimulus to eat them are necessary [19].

As for the low and null associations between the ResE and the other eating behaviors, other studies have shown similar results. For example, a study that included Spanish female undergraduate students also documented a low association between ResE and EmoE (r = 0.34), and a negligible correlation with ExtE (r = 0.17) [5]. However, another study found no correlation with either scale [19]. The authors argued this was unexpected since it had been hypothesized that ResE or dieting elicited a heightened degree of emotionality and externality.

**Figure 2:**
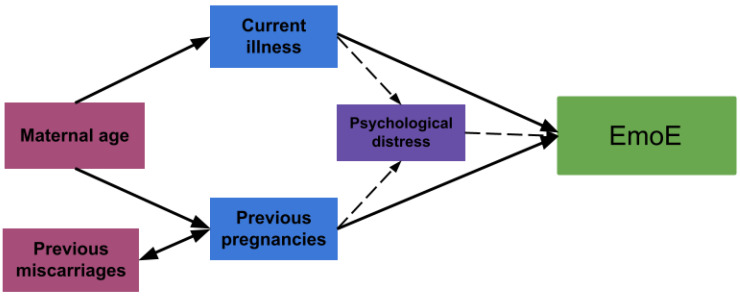
A hypothesis of sociodemographic variables explaining emotional eating behavior.

Regarding correlations with sociodemographic variables and how these may influence eating behaviors, we found that EmoE had a bivariate correlation with maternal age, which was neutralized in the multiple regression model by current illness and a higher number of pregnancies. This is expected, since the latter variables are more likely to be found in older women. It is important to note that EmoE was only minimally explained by current maternal illness, the number of previous pregnancies, and schooling. We suppose the associations with the first two variables could be at least partially mediated by some psychological or emotional factors we did not evaluate. Several studies have described that events or situations like previous pregnancy losses, the demands of caring for older children, and physical illness, are related to the presence of anxiety, stress, and depression during pregnancy [21,22,23]. These psychological traits are in turn associated with EmoE behavior [22,24,25]. Therefore, we may hypothesize that these sociodemographic variables explain EmoE, mediated by psychological variables (Figure 2).

Another interesting finding in our study is the opposite influence of pregestational BMI on two of the measured eating behaviors; it had a positive influence on ResE and a negative one on ExtE. This antagonist influence of BMI on eating behaviors has been previously observed in children, adolescents [15,26], and adults [27]. Notably, we found no association between EmoE and pg-BMI, neither on the bivariate correlations nor on the regression models. This result coincides with reports from Malachowska et al. in adults and Emerson et al. in postpartum women [28,29]. Conversely, in pregnant women, Shakeri et al. found a negative association [9].

Furthermore, in our study, maternal schooling had a positive influence on the three eating behaviors. Likewise, a previous study in adults with diabetes documented that having a high educational level was associated with more restrained, emotional, and external eating behaviors [30].

To discuss these observations, we first need to set out two theoretical arguments, concerning the potential origin and interrelation between the three eating behaviors. The first argument is a vicious cycle of eating behaviors, in which restrained food consumption (ResE) leads to periods of disinhibition or counter-regulation and excessive food consumption [31], triggered by emotional factors (EmoE), such as stress or anxiety, loneliness, deprivation, and low self-esteem [32], and accompanied by external factors (ExtE), like the exposure to food-related cues [33], which, in turn, brings back restrictive behaviors. This cycle may be triggered either by restrictive conduct or by emotional arousal [34]. Putterman and Linden have documented that the periods of disinhibition and overeating are more common when the restrained behavior is motivated by body dissatisfaction or the desire to “improve physical appearance”; and when the restrictive strategies are rigid and “extreme” (i.e., fasting, excluding a whole food group) [31].

**Figure 3:**
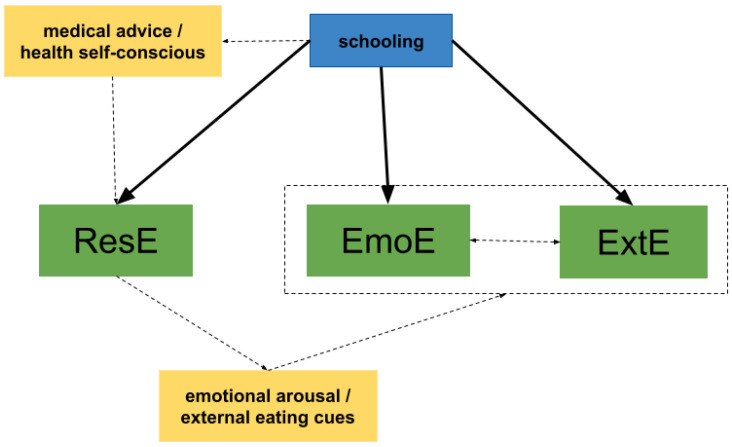
A hypothesis of maternal schooling explaining the restrained, emotional, and external eating behaviors.

Given the cross-sectional design of our study, we may postulate a hypothesis regarding the influence of BMI and education on eating behaviors. With respect to the positive influence of schooling on all three eating behaviors, we could propose that pregnant women with a higher schooling level are more prone to ResE behaviors, possibly in compliance with the traditional medical advice to limit weight gain to a recommended range. This positive influence of education on ResE has been previously observed [35]. In turn, women who practice ResE may undergo disinhibition periods triggered by emotional distress and/or external cues, such as cravings, which are common during pregnancy, possibly reinforcing the cycle of eating behaviors (Figure 3).

A second theoretical argument is that when the restriction is motivated by health and individuals choose flexible strategies to restrict their intake, they are less prone to periods of disinhibition and excessive food consumption, regardless of their emotions and external food cues. An interesting note is that these groups of restrained eaters tend to be older, more educated, and have an external locus of motivation, possibly health professionals advising them to modify their diet [31].

**Figure 4:**
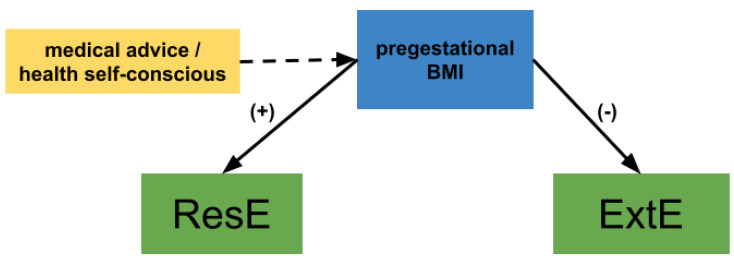
A hypothesis of maternal pregestational BMI explaining the restrained and external eating behaviors.

The antagonistic influence of BMI on ResE and ExtE may be in accordance with the second theoretical argument. It would be the result of weight-centered nutrition interventions offered to pregnant women at the Institute where the study took place and most likely elsewhere. The positive influence of pregestational BMI on ResE may be due to the fact that all women who start pregnancy with a BMI of 25 kg/m^2^ or higher, and/or experience excessive weight gain, are referred to the dietetics service to receive dietary guidance (i.e., caloric restriction). The negative influence on ExtE has been previously observed and, as other authors have discussed, it may be logical to expect an opposite association with BMI since individuals with high ResE scores are already exerting control over their ExtE drive, especially if they are receiving treatment or counseling to manage their eating habits and weight [15,26] (Figure 4).

Our study has some limitations related to criterion validity and test–retest reliability. With respect to criterion validity, in this study, we did not apply other instruments that evaluate eating behaviors, emotions, mental health, or diet indicators in order to assess such validity, since this has already been reported by other authors [4,5,11,14]. Finally, regarding test–retest reliability, we consider that this property cannot be evaluated in pregnant women, since emotional, external, and perhaps even restrictive behaviors can be expected due to the physiologic, psychologic, and emotional adaptations inherent to pregnancy. Such changes in eating behaviors during pregnancy require further research.

## 5. Conclusions

We present a reliable and valid Mexican Spanish version of the DEBQ, useful for being applied to Mexican pregnant women. Our version includes linguistic nuances used in Mexico, which makes it more appropriate for such a population. The psychometric properties of our adaptation confirm its validity and reliability for pregnant women. The three eating behaviors (EmoE, ExtE, and RestE) are differentially associated with maternal sociodemographic and reproductive variables, which partly explain their variation, most notably maternal schooling. Further research would be needed to endorse the validity of our adapted version of the DEBQ in non-pregnant populations.

## Figures and Tables

**Table 1:** Participants’ characteristics (n = 514).

	Median (Q1–Q3)
Age, years	29.7 (25.0–33.8)
Gestational weeks	26.0 (21.0–31.0)
Pregestational BMI, kg/m^2^	25.9 (23.1–30.1)
	n (%)
Number of pregnancies	
First pregnancy	199 (38.7)
2nd or 3rd	216 (42.0)
4th or more	99 (19.3)
One or more previous miscarriages/stillbirths	194 (37.7)
Single pregnancy	486 (94.5)
Living with a current illness	237 (46.1)
Schooling	
Elementary or secondary	137 (26.7)
High school	226 (44.0)
Graduate or postgraduate	151 (29.4)
Occupation	
Stays at home *	354 (68.9)
Works outside home	160 (31.1)
Lives with the baby’s father	
Yes	328 (64.0)
Household welfare	
Medium to high	132 (25.7)
Low	382 (74.3)

* Stays at home category includes students.

**Table 2:** Descriptive statistics for responses to the 33 translated DEBQ items.

Translated Item *	Responses (n = 514)			
	Mean	SD	Minimum	Maximum
Item 1. Cuando ha subido de peso, ¿trata de comer menos…	2.29	1.311	0	5
Item 2. ¿Trata de comer menos…	2.38	1.123	1	5
Item 3. ¿Qué tan seguido rechaza algo…	2.09	1.168	1	5
Item 4. ¿Se fija exactamente en…	2.97	1.168	1	5
Item 5. ¿Come a propósito…	1.60	0.874	1	5
Item 6. Cuando ha comido demasiado…	1.79	1.232	0	5
Item 7. ¿Come usted menos a propósito…	1.75	0.960	1	5
Item 8. ¿Qué tan seguido intenta no comer…	1.97	1.103	1	5
Item 9. ¿Qué tan seguido intenta no cenar…	1.75	0.994	1	5
Item 10. ¿Piensa en su peso cuando…	2.04	1.168	1	5
Item 11. ¿Le dan ganas de comer cuando está…	1.33	1.144	0	5
Item 12. ¿Le dan ganas de comer cuando no…	2.07	1.348	0	5
Item 13. ¿Le dan ganas de comer cuando está…	1.64	1.298	0	5
Item 14. ¿Le dan ganas de comer cuando se …	1.51	1.208	0	5
Item 15. ¿Le dan ganas de comer cuando alguien…	1.06	0.957	0	5
Item 16. ¿Le dan ganas de comer cuando está…	1.22	1.032	0	5
Item 17. ¿Le dan ganas de comer cuando sabe…	1.40	0.806	1	5
Item 18. ¿Le dan ganas de comer cuando está…	1.96	1.151	1	5
Item 19. ¿Le dan ganas de comer cuando las…	1.49	0.841	1	5
Item 20. ¿Le dan ganas de comer cuando está…	0.87	0.876	0	5
Item 21. ¿Le dan ganas de comer cuando está…	1.11	1.035	0	5
Item 22. ¿Le dan ganas de comer cuando se…	1.39	1.212	0	5
Item 23. ¿Le dan ganas de comer cuando está…	1.74	1.318	0	5
Item 24. Si la comida le sabe bien…	2.85	0.975	1	5
Item 25. Si la comida huele…	2.72	0.964	1	5
Item 26. Si ve o huele algo…	3.19	1.002	1	5
Item 27. Si tiene algo delicioso…	3.01	0.998	1	5
Item 28. Si pasa afuera de una panadería…	2.58	1.094	1	5
Item 29. Si pasa afuera de una tiendita…	2.52	0.997	1	5
Item 30. Si ve a otras personas comiendo…	2.15	0.959	1	5
Item 31. ¿Puede resistirse a comer…	3.07	1.031	1	5
Item 32. ¿Come más de lo habitual cuando…	1.65	0.857	1	5
Item 33. Cuando prepara la comida…	2.51	0.992	1	5

* The complete text of the items could not be included due to copyright restrictions of the original scale.

**Table 3:** Item correlations.

Item Number		1	2	3	4	5	6	7	8	9	10	11	12	13	14	15	16	17	18	19	20	21	22	23	24	25	26	27	28	29	30	31	32	33
**1**	r	1	0.637	0.574	0.302	0.387	0.497	0.507	0.465	0.375	0.497	0.077	0.072	0.120	0.159	0.148	0.069	0.132	0.125	0.136	0.119	0.133	0.117	0.087	0.018	−0.019	0.005	−0.051	−0.066	−0.036	0.005	−0.175	0.104	0.001
	*p*		***	***	***	***	***	***	***	***	***	0.083	0.104	0.006	***	0.001	0.117	0.003	0.005	0.002	0.007	0.002	0.008	0.049	0.683	0.660	0.918	0.252	0.135	0.413	0.913	***	0.019	0.973
**2**	r	0.637	1	0.603	0.320	0.352	0.527	0.486	0.498	0.388	0.465	0.115	0.077	0.088	0.136	0.146	0.052	0.089	0.106	0.075	0.074	0.117	0.088	0.108	0.009	0.018	0.051	−0.037	−0.027	−0.008	0.047	−0.179	0.150	0.031
	*p*	***		***	***	***	***	***	***	***	***	0.009	0.080	0.046	0.002	0.001	0.236	0.044	0.016	0.089	0.094	0.008	0.047	0.014	0.835	0.676	0.249	0.403	0.544	0.862	0.286	***	0.001	0.485
**3**	r	0.574	0.603	1	0.474	0.482	0.545	0.538	0.586	0.434	0.623	0.072	0.072	0.098	0.121	0.158	0.070	0.089	0.097	0.091	0.078	0.108	0.097	0.079	−0.030	−0.054	−0.024	−0.133	−0.071	−0.007	0.010	−0.244	0.142	−0.008
	*p*	***	***		***	***	***	***	***	***	***	0.103	0.103	0.026	0.006	***	0.113	0.043	0.028	0.038	0.076	0.015	0.028	0.072	0.504	0.223	0.591	0.003	0.110	0.878	0.824	***	0.001	0.863
**4**	r	0.302	0.320	0.474	1.000	0.237	0.262	0.210	0.318	0.130	0.403	0.040	−0.013	0.052	0.047	0.126	0.056	0.038	−0.026	0.024	0.060	0.079	0.031	−0.006	−0.060	−0.112	−0.050	−0.103	−0.126	−0.092	−0.040	−0.297	−0.017	−0.049
	*p*	***	***	***		***	***	***	***	0.003	***	0.365	0.764	0.237	0.287	0.004	0.209	0.389	0.558	0.595	0.171	0.074	0.484	0.893	0.172	0.011	0.259	0.019	0.004	0.036	0.371	***	0.704	0.266
**5**	r	0.387	0.352	0.482	0.237	1.000	0.442	0.593	0.434	0.364	0.474	0.139	0.206	0.222	0.227	0.223	0.173	0.168	0.217	0.177	0.166	0.224	0.205	0.192	0.107	0.095	0.143	0.056	0.055	0.080	0.120	−0.129	0.246	0.072
	*p*	***	***	***	***		***	***	***	***	***	0.002	***	***	***	***	***	***	***	***	***	***	***	***	0.016	0.032	0.001	0.208	0.215	0.070	0.007	0.004	***	0.101
**6**	r	0.497	0.527	0.545	0.262	0.442	1.000	0.561	0.512	0.454	0.470	0.126	0.135	0.183	0.173	0.195	0.094	0.111	0.157	0.122	0.140	0.152	0.170	0.148	0.037	0.024	0.038	−0.020	−0.010	0.026	0.042	−0.096	0.151	0.036
	*p*	***	***	***	***	***		***	***	***	***	0.004	0.002	***	***	***	0.033	0.012	***	0.006	0.001	0.001	***	0.001	0.405	0.592	0.385	0.654	0.823	0.558	0.348	0.029	0.001	0.420
**7**	r	0.507	0.486	0.538	0.210	0.593	0.561	1.000	0.536	0.494	0.584	0.192	0.203	0.212	0.225	0.258	0.161	0.202	0.216	0.203	0.157	0.190	0.209	0.187	0.134	0.123	0.081	0.004	0.056	0.067	0.085	−0.123	0.224	0.039
	*p*	***	***	***	***	***	***		***	***	***	***	***	***	***	***	***	***	***	***	***	***	***	***	0.002	0.005	0.066	0.935	0.209	0.129	0.055	0.005	***	0.384
**8**	r	0.465	0.497	0.586	0.318	0.434	0.512	0.536	1.000	0.561	0.534	0.072	0.051	0.085	0.103	0.144	0.040	0.050	0.086	0.075	0.073	0.084	0.093	0.072	−0.017	−0.055	−0.088	−0.034	−0.006	−0.018	−0.016	−0.124	0.103	−0.045
	*p*	***	***	***	***	***	***	***		***	***	0.101	0.248	0.055	0.020	0.001	0.364	0.262	0.051	0.090	0.097	0.056	0.035	0.104	0.704	0.211	0.046	0.445	0.884	0.676	0.721	0.005	0.020	0.311
**9**	r	0.375	0.388	0.434	0.130	0.364	0.454	0.494	0.561	1.000	0.478	0.069	0.070	0.102	0.058	0.116	0.045	0.061	0.083	0.048	−0.001	0.054	0.053	0.024	0.037	0.047	−0.057	−0.049	−0.038	−0.034	−0.047	−0.069	0.115	−0.043
	*p*	***	***	***	0.003	***	***	***	***		***	0.120	0.112	0.021	0.186	0.009	0.312	0.164	0.061	0.280	0.989	0.222	0.227	0.593	0.398	0.284	0.201	0.267	0.391	0.444	0.292	0.121	0.009	0.331
**10**	r	0.497	0.465	0.623	0.403	0.474	0.470	0.584	0.534	0.478	1.000	0.122	0.130	0.182	0.172	0.194	0.077	0.118	0.143	0.135	0.095	0.114	0.150	0.132	0.021	0.019	0.077	−0.005	0.039	0.081	0.019	−0.187	0.136	0.013
	*p*	***	***	***	***	***	***	***	***	***		0.006	0.003	***	***	***	0.081	0.007	0.001	0.002	0.031	0.010	0.001	0.003	0.638	0.661	0.082	0.918	0.376	0.068	0.664	***	0.002	0.763
**11**	r	0.077	0.115	0.072	0.040	0.139	0.126	0.192	0.072	0.069	0.122	1.000	0.509	0.597	0.560	0.547	0.690	0.427	0.471	0.496	0.465	0.455	0.534	0.498	0.300	0.315	0.208	0.207	0.269	0.220	0.273	0.035	0.300	0.157
	*p*	0.083	0.009	0.103	0.365	0.002	0.004	***	0.101	0.120	0.006		***	***	***	***	***	***	***	***	***	***	***	***	***	***	***	***	***	***	***	0.430	***	***
**12**	r	0.072	0.077	0.072	−0.013	0.206	0.135	0.203	0.051	0.070	0.130	0.509	1.000	0.585	0.570	0.423	0.484	0.376	0.545	0.462	0.372	0.405	0.488	0.673	0.396	0.414	0.372	0.331	0.361	0.326	0.329	0.071	0.343	0.242
	*p*	0.104	0.080	0.103	0.764	***	0.002	***	0.248	0.112	0.003	***		***	***	***	***	***	***	***	***	***	***	***	***	***	***	***	***	***	***	0.109	***	***
**13**	r	0.120	0.088	0.098	0.052	0.222	0.183	0.212	0.085	0.102	0.182	0.597	0.585	1.000	0.717	0.552	0.652	0.464	0.637	0.557	0.481	0.530	0.686	0.554	0.257	0.305	0.235	0.240	0.251	0.239	0.230	0.024	0.322	0.192
	*p*	0.006	0.046	0.026	0.237	***	***	***	0.055	0.021	***	***	***		***	***	***	***	***	***	***	***	***	***	***	***	***	***	***	***	***	0.583	***	***
**14**	r	0.159	0.136	0.121	0.047	0.227	0.173	0.225	0.103	0.058	0.172	0.560	0.570	0.717	1.000	0.627	0.632	0.479	0.566	0.545	0.525	0.576	0.682	0.588	0.280	0.298	0.278	0.271	0.263	0.279	0.273	0.034	0.308	0.223
	*p*	***	0.002	0.006	0.287	***	***	***	0.020	0.186	***	***	***	***		***	***	***	***	***	***	***	***	***	***	***	***	***	***	***	***	0.445	***	***
**15**	r	0.148	0.146	0.158	0.126	0.223	0.195	0.258	0.144	0.116	0.194	0.547	0.423	0.552	0.627	1.000	0.712	0.471	0.429	0.490	0.632	0.650	0.589	0.466	0.190	0.207	0.203	0.169	0.187	0.197	0.176	0.013	0.310	0.139
	*p*	0.001	0.001	***	0.004	***	***	***	0.001	0.009	***	***	***	***	***		***	***	***	***	***	***	***	***	***	***	***	***	***	***	***	0.768	***	0.002
**16**	r	0.069	0.052	0.070	0.056	0.173	0.094	0.161	0.040	0.045	0.077	0.690	0.484	0.652	0.632	0.712	1.000	0.487	0.516	0.564	0.608	0.607	0.636	0.488	0.256	0.278	0.218	0.202	0.270	0.243	0.250	0.075	0.261	0.202
	*p*	0.117	0.236	0.113	0.209	***	0.033	***	0.364	0.312	0.081	***	***	***	***	***		***	***	***	***	***	***	***	***	***	***	***	***	***	***	0.088	***	***
**17**	r	0.132	0.089	0.089	0.038	0.168	0.111	0.202	0.050	0.061	0.118	0.427	0.376	0.464	0.479	0.471	0.487	1.000	0.530	0.654	0.485	0.482	0.516	0.414	0.239	0.257	0.198	0.189	0.224	0.149	0.258	0.008	0.364	0.180
	*p*	0.003	0.044	0.043	0.389	***	0.012	***	0.262	0.164	0.007	***	***	***	***	***	***		***	***	***	***	***	***	***	***	***	***	***	0.001	***	0.849	***	***
**18**	r	0.125	0.106	0.097	−0.026	0.217	0.157	0.216	0.086	0.083	0.143	0.471	0.545	0.637	0.566	0.429	0.516	0.530	1.000	0.679	0.426	0.458	0.603	0.582	0.297	0.322	0.307	0.289	0.311	0.270	0.273	0.009	0.363	0.195
	*p*	0.005	0.016	0.028	0.558	***	***	***	0.051	0.061	0.001	***	***	***	***	***	***	***		***	***	***	***	***	***	***	***	***	***	***	***	0.834	***	***
**19**	r	0.136	0.075	0.091	0.024	0.177	0.122	0.203	0.075	0.048	0.135	0.496	0.462	0.557	0.545	0.490	0.564	0.654	0.679	1.000	0.513	0.550	0.608	0.527	0.323	0.348	0.284	0.251	0.292	0.250	0.335	0.057	0.381	0.253
	*p*	0.002	0.089	0.038	0.595	***	0.006	***	0.090	0.280	0.002	***	***	***	***	***	***	***	***		***	***	***	***	***	***	***	***	***	***	***	0.196	***	***
**20**	r	0.119	0.074	0.078	0.060	0.166	0.140	0.157	0.073	−0.001	0.095	0.465	0.372	0.481	0.525	0.632	0.608	0.485	0.426	0.513	1.000	0.727	0.640	0.469	0.267	0.288	0.243	0.282	0.240	0.212	0.211	0.054	0.298	0.244
	*p*	0.007	0.094	0.076	0.171	***	0.001	***	0.097	0.989	0.031	***	***	***	***	***	***	***	***	***		***	***	***	***	***	***	***	***	***	***	0.220	***	***
**21**	r	0.133	0.117	0.108	0.079	0.224	0.152	0.190	0.084	0.054	0.114	0.455	0.405	0.530	0.576	0.650	0.607	0.482	0.458	0.550	0.727	1.000	0.749	0.514	0.296	0.289	0.249	0.254	0.226	0.206	0.213	0.014	0.275	0.243
	*p*	0.002	0.008	0.015	0.074	***	0.001	***	0.056	0.222	0.010	***	***	***	***	***	***	***	***	***	***		***	***	***	***	***	***	***	***	***	0.747	***	***
**22**	r	0.117	0.088	0.097	0.031	0.205	0.170	0.209	0.093	0.053	0.150	0.534	0.488	0.686	0.682	0.589	0.636	0.516	0.603	0.608	0.640	0.749	1.000	0.604	0.304	0.328	0.276	0.285	0.282	0.225	0.283	0.025	0.316	0.214
	*p*	0.008	0.047	0.028	0.484	***	***	***	0.035	0.227	0.001	***	***	***	***	***	***	***	***	***	***	***		***	***	***	***	***	***	***	***	0.566	***	***
**23**	r	0.087	0.108	0.079	−0.006	0.192	0.148	0.187	0.072	0.024	0.132	0.498	0.673	0.554	0.588	0.466	0.488	0.414	0.582	0.527	0.469	0.514	0.604	1.000	0.393	0.429	0.380	0.344	0.331	0.331	0.315	0.067	0.402	0.252
	*p*	0.049	0.014	0.072	0.893	***	0.001	***	0.104	0.593	0.003	***	***	***	***	***	***	***	***	***	***	***	***		***	***	***	***	***	***	***	0.128	***	***
**24**	r	0.018	0.009	−0.030	−0.060	0.107	0.037	0.134	−0.017	0.037	0.021	0.300	0.396	0.257	0.280	0.190	0.256	0.239	0.297	0.323	0.267	0.296	0.304	0.393	1.000	0.798	0.538	0.535	0.399	0.327	0.393	0.129	0.363	0.364
	*p*	0.683	0.835	0.504	0.172	0.016	0.405	0.002	0.704	0.398	0.638	***	***	***	***	***	***	***	***	***	***	***	***	***		***	***	***	***	***	***	0.003	***	***
**25**	r	−0.019	0.018	−0.054	−0.112	0.095	0.024	0.123	−0.055	0.047	0.019	0.315	0.414	0.305	0.298	0.207	0.278	0.257	0.322	0.348	0.288	0.289	0.328	0.429	0.798	1.000	0.557	0.498	0.400	0.348	0.445	0.118	0.420	0.380
	*p*	0.660	0.676	0.223	0.011	0.032	0.592	0.005	0.211	0.284	0.661	***	***	***	***	***	***	***	***	***	***	***	***	***	***		***	***	***	***	***	0.007	***	***
**26**	r	0.005	0.051	−0.024	−0.050	0.143	0.038	0.081	−0.088	−0.057	0.077	0.208	0.372	0.235	0.278	0.203	0.218	0.198	0.307	0.284	0.243	0.249	0.276	0.380	0.538	0.557	1.000	0.597	0.510	0.470	0.447	0.095	0.328	0.401
	*p*	0.918	0.249	0.591	0.259	0.001	0.385	0.066	0.046	0.201	0.082	***	***	***	***	***	***	***	***	***	***	***	***	***	***	***		***	***	***	***	0.032	***	***
**27**	r	−0.051	−0.037	−0.133	−0.103	0.056	−0.020	0.004	−0.034	−0.049	−0.005	0.207	0.331	0.240	0.271	0.169	0.202	0.189	0.289	0.251	0.282	0.254	0.285	0.344	0.535	0.498	0.597	1.000	0.480	0.500	0.421	0.220	0.346	0.432
	*p*	0.252	0.403	0.003	0.019	0.208	0.654	0.935	0.445	0.267	0.918	***	***	***	***	***	***	***	***	***	***	***	***	***	***	***	***		***	***	***	***	***	***
**28**	r	−0.066	−0.027	−0.071	−0.126	0.055	−0.010	0.056	−0.006	−0.038	0.039	0.269	0.361	0.251	0.263	0.187	0.270	0.224	0.311	0.292	0.240	0.226	0.282	0.331	0.399	0.400	0.510	0.480	1.000	0.678	0.409	0.091	0.273	0.325
	*p*	0.135	0.544	0.110	0.004	0.215	0.823	0.209	0.884	0.391	0.376	***	***	***	***	***	***	***	***	***	***	***	***	***	***	***	***	***		***	***	0.040	***	***
**29**	r	−0.036	−0.008	−0.007	−0.092	0.080	0.026	0.067	−0.018	−0.034	0.081	0.220	0.326	0.239	0.279	0.197	0.243	0.149	0.270	0.250	0.212	0.206	0.225	0.331	0.327	0.348	0.470	0.500	0.678	1.000	0.428	0.137	0.300	0.307
	*p*	0.413	0.862	0.878	0.036	0.070	0.558	0.129	0.676	0.444	0.068	***	***	***	***	***	***	0.001	***	***	***	***	***	***	***	***	***	***	***		***	0.002	***	***
**30**	r	0.005	0.047	0.010	−0.040	0.120	0.042	0.085	−0.016	−0.047	0.019	0.273	0.329	0.230	0.273	0.176	0.250	0.258	0.273	0.335	0.211	0.213	0.283	0.315	0.393	0.445	0.447	0.421	0.409	0.428	1.000	0.198	0.552	0.441
	*p*	0.913	0.286	0.824	0.371	0.007	0.348	0.055	0.721	0.292	0.664	***	***	***	***	***	***	***	***	***	***	***	***	***	***	***	***	***	***	***		***	***	***
**31**	r	−0.175	−0.179	−0.244	−0.297	−0.129	−0.096	−0.123	−0.124	−0.069	−0.187	0.035	0.071	0.024	0.034	0.013	0.075	0.008	0.009	0.057	0.054	0.014	0.025	0.067	0.129	0.118	0.095	0.220	0.091	0.137	0.198	1.000	0.133	0.081
	*p*	***	***	***	***	0.004	0.029	0.005	0.005	0.121	***	0.430	0.109	0.583	0.445	0.768	0.088	0.849	0.834	0.196	0.220	0.747	0.566	0.128	0.003	0.007	0.032	***	0.040	0.002	***		0.003	0.066
**32**	r	0.104	0.150	0.142	−0.017	0.246	0.151	0.224	0.103	0.115	0.136	0.300	0.343	0.322	0.308	0.310	0.261	0.364	0.363	0.381	0.298	0.275	0.316	0.402	0.363	0.420	0.328	0.346	0.273	0.300	0.552	0.133	1.000	0.315
	*p*	0.019	0.001	0.001	0.704	***	0.001	***	0.020	0.009	0.002	***	***	***	***	***	***	***	***	***	***	***	***	***	***	***	***	***	***	***	***	0.003		***
**33**	r	0.001	0.031	−0.008	−0.049	0.072	0.036	0.039	−0.045	−0.043	0.013	0.157	0.242	0.192	0.223	0.139	0.202	0.180	0.195	0.253	0.244	0.243	0.214	0.252	0.364	0.380	0.401	0.432	0.325	0.307	0.441	0.081	0.315	1.000
	*p*	0.973	0.485	0.863	0.266	0.101	0.420	0.384	0.311	0.331	0.763	***	***	***	***	0.002	***	***	***	***	***	***	***	***	***	***	***	***	***	***	***	0.066	***	

r = Spearman correlation value; *p* = significance value; *** *p* < 0.001.

**Table 4:** Rotated component matrix.

Item *	Component		
	1	2	3
16. ¿Le dan ganas de comer cuando…	0.836		
22. ¿Le dan ganas de comer cuando…	0.834		
15. ¿Le dan ganas de comer cuando…	0.813		
21. ¿Le dan ganas de comer cuando…	0.802		
19. ¿Le dan ganas de comer cuando…	0.800		
14. ¿Le dan ganas de comer cuando…	0.790		
13. ¿Le dan ganas de comer cuando…	0.788		
20. ¿Le dan ganas de comer cuando…	0.756		
17. ¿Le dan ganas de comer cuando…	0.741		
11. ¿Le dan ganas de comer cuando…	0.734		
18. ¿Le dan ganas de comer cuando…	0.732		
3. ¿Qué tan seguido rechaza…		0.806	
7. ¿Come usted menos…		0.778	
8. ¿Qué tan seguido intenta…		0.754	
2. ¿Trata de comer menos…		0.752	
10. ¿Piensa en su peso…		0.750	
6. Cuando ha comido demasiado…		0.743	
1. Cuando ha subido de peso…		0.734	
9. ¿Qué tan seguido intenta…		0.656	
5. ¿Come a propósito…		0.628	
26. Si se ve o huele algo…			0.803
27. Si tiene algo delicioso…			0.777
24. Si la comida le sabe bien…			0.765
25. Si la comida huele…			0.758
28. Si pasa afuera de…			0.742
29. Si pasa afuera de…			0.703

* The complete text of the items could not be included due to copyright restrictions of the original scale.

**Table 5:** Correlations between eating behaviors and psychosocial factors.

	ResE	EmoE	ExtE	Maternal Age	pg-BMI	Number of Pregnancies	Previous Miscarriage	Type of Pregnancy	GW	Current Illness	Schooling	Occupation	Lives with Baby’s Father	Household Welfare
ResE	-	**0.205** **(0.000)**	0.015 (0.736)	**0.323** **(0.000)**	**0.419** **(0.000)**	**0.098** **(0.026)**	0.073 (0.097)	0.006 (0.898)	−0.049 (0.272)	0.084 (0.057)	**0.176** **(0.000)**	**0.103** **(0.019)**	0.043 (0.331)	**0.099** **(0.025)**
EmoE		-	**0.429** **(0.000)**	**0.113** **(0.010)**	0.016 (0.726)	−0.005 (0.915)	0.021 (0.636)	−0.046 (0.301)	−0.017 (0.693)	0.061 (0.164)	**0.265** **(0.000)**	0.022 (0.620)	0.017 (0.695)	0.070 (0.113)
ExtE			-	−0.020 (0.657)	**−0.087** **(0.050)**	−0.015 (0.737)	0.042 (0.344)	0.013 (0.766)	**0.088** **(0.047)**	0.023 (0.609)	**0.107** **(0.000)**	−0.009 (0.842)	0.022 (0.614	0.015 (0.740)
Maternal Age				-	**0.293** **(0.000)**	**0.255** **(0.000)**	**0.193** **(0.000)**	−0.059 (0.183)	−0.059 (0.183)	**0.103** **(0.020)**	**0.163** **(0.000)**	**0.161** **(0.000)**	**0.148** **(0.001)**	0.087 (0.050)
pg-BMI					-	**0.106** **(0.016)**	0.047 (0.291)	−0.002 (0.957)	−0.015 (0.738)	0.037 (0.407)	−0.059 (0.181)	0.018 (0.688)	0.049 (0.074)	0.021 (0.633)
Number of pregnancies						-	**0.687** **(0.000)**	−0.019 (0.670)	−0.006 (0.894)	−0.064 (0.150)	**−0.207** **(0.000)**	**−0.168** **(0.000)**	**0.247** **(0.000)**	**−0.111** **(0.012)**
Previous miscarriage							-	−0.063 (0.153)	**−0.107** **(0.015)**	−0.036 (0.417)	−0.013 (0.763)	−0.038 (0.389)	**0.119** **(0.007)**	−0.035 (0.427)
Type of pregnancy								-	0.070 (0.114)	**−0.170** **(0.000)**	−0.059 (0.179)	−0.032 (0.472)	**0.092** **(0.038)**	**−0.102** **(0.021)**
GW									-	−0.039 (0.376)	**−0.153** **(0.001)**	−0.083 (0.060)	−0.017 (0.704)	−0.069 (0.120)
Current illness										-	0.049 (0.272)	0.044 (0.319)	−0.026 (0.552)	**0.609** **(0.000)**
Schooling											-	**0.268** **(0.000)**	**0.098** **(0.027)**	**0.225** **(0.000)**
Occupation												-	**−0.211** **(0.000)**	**0.163** **(0.000)**
Lives with baby’s father													-	0.002 (0.961)
Household welfare														-

Spearman’s rho correlation. Bold numbers = significant at the 0.01 level. pg-BMI = pregestational BMI. GW = gestational weeks. Number of pregnancies = three categories (1 = first pregnancy, 2 = second or third pregnancy, 3 = fourth or more pregnancies). Previous miscarriages/stillbirths: 0 = no, 1 = yes. Type of pregnancy: 1 = single, 2 = multiple (two or more babies). Current illness: 0 = no, 1 = yes. Occupation: 1 = stays at home, 2 = works away from home. Lives with baby’s father: 0 = no, 1 = yes. Household welfare:1 = low, 2 = high.

**Table 6:** Linear regression models.

Restrained Eating		Emotional Eating		External Eating	
Predictors	𝛽 * (95% CI), *p*-Value	Predictors	𝛽 * (95% CI), *p*-Value	Predictors	𝛽 * (95% CI), *p*-Value
Schooling	0.134 (0.063, 0.205), <0.001	Schooling	0.220 (0.140, 0.300), <0.001	Schooling	0.104 (0.029, 0.179), 0.007
pg-BMI	0.045 (0.033, 0.56), <0.001	Current illness **	0.173 (0.030, 0.316), 0.018	Gestational weeks	0.010 (0.000, 0.021), 0.060
Age	0.030 (0.018, 0.042), <0.001	Number of pregnancies	0.085 (−0.014, 0.184), 0.090	pg-BMI	−0.013 (−0.025, −0.002), 0.027
R^2^ (adjusted R^2^)	0.205 (0.200)	R^2^ (adjusted R^2^)	0.065 (0.059)	R^2^ (adjusted R^2^)	0.030 (0.024)

Linear regressions using the backward variable selection procedure. The same variables were included in the three models. Variables not significant in any model were: previous miscarriage, occupation, lives with the baby’s father, and household welfare. * unstandardized 𝛽, ** yes = 1, no = 0. pg-BMI = pregestational BMI.

## Data Availability

The data presented in this study are available on request from the corresponding author. The data are not publicly available due to copyright restrictions of the original scale.
